# *Lactobacillus reuteri* BM53-1 Produces a Compound That Inhibits Sticky Glucan Synthesis by *Streptococcus mutans*

**DOI:** 10.3390/microorganisms9071390

**Published:** 2021-06-27

**Authors:** Masafumi Noda, Naho Sugihara, Yoshimi Sugimoto, Ikue Hayashi, Sachiko Sugimoto, Narandalai Danshiitsoodol, Masanori Sugiyama

**Affiliations:** 1 Department of Probiotic Science for Preventive Medicine, Graduate School of Biomedical and Health Sciences, Hiroshima University, Hiroshima 734-8551, Japan; bel@hiroshima-u.ac.jp (M.N.); m161349@hiroshima-u.ac.jp (N.S.); m106564@hiroshima-u.ac.jp (Y.S.); naraa@hiroshima-u.ac.jp (N.D.); 2 Central Research Laboratory, Graduate School of Biomedical and Health Sciences, Hiroshima University, Hiroshima 734-8551, Japan; ikue@hiroshima-u.ac.jp; 3 Department of Pharmacognosy, Graduate School of Biomedical and Health Sciences, Hiroshima University, Hiroshima 734-8551, Japan; ssugimot@hiroshima-u.ac.jp

**Keywords:** biofilm, lactic acid bacteria (LAB), *Streptococcus mutans*, *Lactobacillus reuteri*

## Abstract

Cariogenic bacteria, such as *Streptococcus* (*S.*) *mutans* and *S. sobrinus*, produce insoluble and sticky glucans as a biofilm material. The present study demonstrates that a lactic acid bacterium (LAB) named BM53-1 produces a substance that inhibits the sticky glucan synthesis. The BM53-1 strain was isolated from a flower of *Actinidia polygama* and identified as *Lactobacillus reuteri*. The substance that inhibits sticky glucan synthesis does not exhibit antibacterial activity against *S. mutans*. The cariogenic *S. mutans* produces glucans under the control of three glucosyltransferase (GTF) enzymes, named GtfB, GtfC, and GtfD. Although GtfB and GtfC produce insoluble glucans, GtfD forms soluble glucans. Through quantitative reverse-transcriptional (qRT)-PCR analysis, it was revealed that the BM53-1-derived glucan-production inhibitor (GI) enhances the transcriptions of *gtfB* and *gtfC* genes 2- to 7-fold at the early stage of cultivation. However, that of *gtfD* was not enhanced in the presence of the GI, indicating that the glucan stickiness produced by *S. mutans* was significantly weaker in the presence of the GI. Our result demonstrates that *Lb. reuteri* BM53-1 is useful to prevent dental caries.

## 1. Introduction

Dental caries is caused by local and continuous acid production from carbohydrates by oral bacteria [1,2]. The bacterial infection causes demineralization, represented by loss of calcium, phosphate, and other ions from the tooth enamel. On the other hand, the lost minerals are absorbed into the tooth enamel again when the oral pH is neutralized by the buffering effect of saliva. This recovery process is called remineralization. The demineralization and remineralization steps on dental enamel are naturally cycled in the oral cavity; however, when the balance between these two steps is biased toward mineral loss, dental caries (tooth decay) occurs [3].

The tooth surface is coated by a thin layer, called a pellicle, that consists of many proteins contained in saliva [4,5,6,7]. *Streptococcus* (*S.*) *mutans* and *S. sobrinus*, which are known as cariogenic bacteria, produce insoluble and adhesive glucans from sucrose in the oral cavity [8,9,10]. These bacteria adhere to the tooth surface through the van der Waals force between the early colonizers of dental plaque that nonspecifically adhere to the pellicle layer [11,12]. Both species also recognize the saccharide residue of the pellicle layer as an adhesive ligand. When *S. mutans* and *S. sobrinus* harboring glucosyltransferase (GTF) produce insoluble glucans on the tooth surface, the glucans pile up with other incorporated oral bacteria and form the firm and stable structure called a biofilm [9,12]. Since the biofilm structure resists salivary washing and physical removal such as tooth brushing, the inner niche is separated from the salivary buffering effect, and inner bacteria can stably grow. Therefore, the partial pH decrease is continued in the highly structured biofilm, resulting in dental caries.

Since probiotics such as LAB strains are nonpathogenic, their importance in the food industry has attracted considerable attention [13]. Lactic acid bacterial (LAB) strains have traditionally been used to produce fermented foods, which have health benefits for humans, such as intestinal homeostasis, anti-allergic properties, and oral health-care control [14,15,16,17,18,19,20,21]. We have isolated many kinds of plant-derived LAB strains from fruits, vegetables, flowers, and medicinal plants. It has been reported that some LAB strains stored in the library exhibit preventive and presymptomatic medicinal properties [22,23,24,25,26]. Furthermore, we have found the plant-derived-LAB strains that produce anti-bacterial substances effective against cariogenic *S. mutans* [27] and *Porphyromonas gingivalis*, which causes periodontal inflammatory diseases [28]. In the present study, we inferred the inhibitory mechanism and molecular characteristics of the BM53-1-derived substance that inhibits the synthesis of sticky glucans (glucan-synthesizing inhibitor; GI).

## 2. Materials and Methods

### 2.1. Bacterial Strains and Culture Conditions

*Streptococcus mutans* MT8148R is a streptomycin (1500 μg/mL)-resistant strain generated from the MT8148 strain, which was isolated at Osaka University Dental Hospital and was laboratory-maintained in the Department of Dental Research, the National Institute of Health, Tokyo, Japan [29,30,31]. *Streptococcus mutans* MT8148R and *S. sobrinus* ATCC27607 (human origin strain) were used as cariogenic bacteria that produce glucans as a biofilm material. de Man, Rogosa, and Sharpe (MRS) broth (Merck KGaA, Darmstadt, Germany) was used for LAB cultivation. Brain heart infusion (BHI) broth (Difco, Franklin Lakes, NJ, USA) was used to grow *Streptococcus* strains. When necessary, 1 mg/mL streptomycin (Wako Pure Chemical Industries, Ltd., Osaka, Japan), 1.0% (*w/v*) sucrose (Wako Pure Chemical Industries, Ltd., Osaka, Japan) and 1.5% (*w/v*) agar (Wako Pure Chemical Industries, Ltd., Osaka, Japan) were added to each medium. To produce the GI, *Lactobacillus* (*Lb.*) *reuteri* BM53-1 was cultured in a carrot juice medium consisting of carrot juice and 1% (*w/v*) sake lees (kindly provided by SAKURAO Brewery and Distillery Co., Ltd., Hiroshima, Japan). The carrot juice medium was sterilized at 105 °C for 5 min before inoculation. All strains were stored at −80 °C until use as frozen stock in each medium containing 16.5% (*v/v*) glycerol (Wako Pure Chemical Industries, Ltd., Osaka, Japan).

### 2.2. Assay Method for Anti-Glucan-Forming Activity

The amount of glucans produced by *S. mutans* MT8148R and *S. sobrinus* ATCC27607 was calculated based on the theory reported by Pedersen [32], in which the dried glucan mass correlates with the amount of crystal violet absorbed with the glucans, that is, a 50 μL portion of the assay sample was added into each 96-well flat-bottomed microtiter plate with a 50 μL portion of the 2× BHI broth containing 2% (*w/v*) sucrose and *S. mutans* or *S. sobrinus* cells (adjusted to 6–9 × 10^6^ colony forming unit (CFU) /mL). The sample was adjusted to pH 7–8 and passed through a 0.22 μm pore-size filter (DISMIC disposable membrane filter unit, Advantec Toyo Kaisha, Ltd., Tokyo, Japan) beforehand. After incubation at 37 °C for 24 h without shaking, the glucans formed on the bottom of the plate were rinsed with distilled water three times and then dried. A 100 μL portion of 0.1% (*w/v*) crystal violet (Wako Pure Chemical Industries, Ltd., Osaka, Japan) was added and incubated for 15 min. After staining, the glucans were rinsed with distilled water three times and then dried. The absorbed crystal violet was extracted to a 100 μL portion of 95% (*v/v*) EtOH (Wako Pure Chemical Industries, Ltd., Osaka, Japan) under a shaking condition at 4 °C for 20 min. A 90 μL aliquot of the extract was transferred to the new 96-well microtiter plate, and *A*_595_ was monitored using a microplate absorbance reader (iMark, Bio-Rad Laboratories, Inc., Hercules, CA, USA)

To screen for LAB strains that exhibit glucan-synthesizing inhibitor activity from our established plant-derived LAB library, each LAB cell suspension (6–9 × 10^6^ CFU/mL) and MRS medium was used instead of a filtrated sample and BHI medium, respectively.

### 2.3. Chromosomal DNA Preparation and LAB Strain Identification

The chromosomal DNA from LAB cells was isolated using a DNA Zol reagent (Invitrogen, Carlsbad, CA, USA) according to the manufacturer’s protocol. To identify the LAB isolates, the entire 16S ribosomal DNA (rDNA) sequence of each strain was determined as described previously [33,34,35] and compared with that of typical LABs registered on the DNA Data Bank of Japan (DDBJ) website (http://www.ddbj.nig.ac.jp (accessed on 30 July 2010)). The nucleotide sequences were determined by the ABI PRIZM 310 genetic analyzer using the BigDye Terminator v1.1 Cycle Sequencing Kit (Applied Biosystems, Foster City, CA, USA) according to the manufacturer’s protocol. The nucleotide sequence data was analyzed using ATGC software (GENETYX Corporation, Tokyo, Japan).

The homology search analysis was performed using the BLAST algorithm [36]. Through analysis of the 16S rDNA sequence alignment using the ClustalW program (http://clustalw.ddbj.nig.ac.jp/index.php?lang=ja (accessed on 30 July 2010)), the LAB species names were determined.

### 2.4. Preparation of the GI Fraction from the BM53-1 Strain Culture Broth

To obtain the GI fraction produced by the BM53-1 strain, the strain was cultured in carrot juice as a medium. After 18 h of cultivation at 37 °C, the pH of the culture was adjusted to 7.0 by adding NaOH solution. The culture broth was centrifuged at 8000× *g* for 40 min at 4 °C, and the resulting supernatant was filtrated through a 2 μm pore-size membrane filter (Advantec Toyo Kaisha, Ltd., Tokyo, Japan). The filtrate was concentrated to one-twentieth of the original volume and dialyzed against distilled water using an Amicon Ultracel-10K ultrafiltration device (Merck Millipore, Ltd., Carrigtwohill, Co. Cork, Ireland). After removing debris from the concentrate by centrifugation at 13,000× *g* for 10 min at 4 °C, the resulting supernatant was filtrated with a 0.22 μm pore-size membrane filter (DISMIC disposable membrane filter unit, Advantec Toyo Kaisha, Ltd., Tokyo, Japan) and stored at 4 °C until use.

### 2.5. Scanning Electron Microscopy (SEM) Analysis

The *S. mutans* cells were washed twice with 10 mM phosphate buffered saline (PBS, 0.35 g/L of NaH_2_PO_4_, 1.28 g/L of Na_2_HPO_4_, and 8 g/L of NaCl, pH 7.3) and fixed with 2% (*w/v*) glutaraldehyde at 4 °C for overnight. After washing the fixed cell well with PBS three times, the specimen was dehydrated with increasing concentration of ethanol and 100% ethanol twice for 10 min. Then these were followed by subjecting to critical point drying by use of *t*-butyl alcohol. Dried samples were used for SEM analysis.

The SEM images were captured at a magnification of ×12,000 with an acceleration voltage of 30 kV using a Hitachi S-5200 scanning electron microscope (Hitachi High-Technologies Corp., Tokyo, Japan) after gold particles coating.

### 2.6. Acute Oral Toxicity Test

After, an acute oral toxicity test of the BM53-1 cells was performed at Japan Food Research Laboratories (Tokyo, Japan) in accordance with the OECD Guidelines for the Testing of Chemicals (Guidelines 420, 2001). The experimental procedure was approved by the ethics committee established within the company. Slc:Wistar/ST male rats (5 weeks old) were purchased from Japan SLC, Inc. (Shizuoka, Japan). The rats were divided into two groups of five rats each and housed in polycarbonate cages in a temperature-controlled room (23 ± 3 °C) with 12 h light–dark cycles. Rats had free access to drinking water and a Labo MR stock diet (Nosan Co., Yokohama, Japan).

After a 1-week acclimation period, one group was assigned to be a reference group and the other a LAB-fed group. After cultivation, the LAB cells were collected and resuspended into the water for injection (100 mg LAB cells/mL), and a 20 mL/kg dose of the cell suspension was administered to the LAB-fed group of rats orally every day using a sterile stomach tube. In the reference group, instead of the cell suspension, a water for injection was administered. During the 14-day experiment period, the activity, behavior, and health status of the rats were recorded every day, and their body weight was measured at 1, 7, and 14 days. The rats were euthanized after the experimental period, and some extracted organs were analyzed histologically. Student’s or Welch’s *t*-test was used to analyze the body weight difference among rats using the IBM SPSS Statistics 22 (IBM Japan, Tokyo, Japan). The same research was also carried out using female rats.

### 2.7. Bacterial Reverse Mutation Test (Ames Test)

The bacterial reverse mutation on the BM53-1 heat-killed cell was performed at the BoZo Research Center, Inc. (Tokyo, Japan) under Good Laboratory Practice (GLP) conditions. As a positive control of mutagens, 2-(2-furyl)-3-(5-nitro-2-furyl) acrylamide (AF-2, Wako Pure Chemical Industries, Ltd., Osaka, Japan), sodium azide (SAZ, Wako Pure Chemical Industries, Ltd., Osaka, Japan), 2-aminoanthracene (2-AA, Wako Pure Chemical Industries, Ltd.), 2-methoxy-6-chloro-9-[3-(2-chloroethyl)-aminopropylamino] acridine HCl (ICR-191, Polyscience, Inc., Warrington, PA, USA), and benzo[α]pyrene (B[α]P, Wako Pure Chemical Industries, Ltd., Osaka, Japan) were dissolved into dimethyl sulfoxide (DMSO, Wako Pure Chemical Industries, Ltd., Osaka, Japan) and used for the assay. *Salmonella enterica* subsp. *enterica* serovar Typhimurium strains TA98 and TA1537 were used to evaluate a frameshift mutation, whereas strains TA100 and TA153 and *Escherichia* (*E.*) *coli* WP2uvrA were used to detect base-pair substitutions.

One hundred–microliter aliquots of DMSO (negative control), each mutagen (positive control) solution, and the BM53-1 cell suspension were added to 500 μL of a 100 mM phosphate buffer (pH 7.4). If the metabolic activation was evaluated, an S9 mixture (Kikkoman Biochemifa Co., Ltd., Tokyo, Japan) supplemented with cofactor-I (Oriental Yeast Co., Ltd., Tokyo, Japan) was used instead of the phosphate buffer.

After adding 100 μL of *Salmonella* or *E. coli* cell suspensions, the assay mixtures were pre-incubated at 37 °C for 20 min with shaking, followed by adding 2 mL of preheated (45 °C) top agar (0.6% *w/v* agar, 0.6% *w/v* NaCl, 0.5 mM D-biotin, 0.5 mM L-histidine, and 0.5 mM L-tryptophan). The assay mixture was immediately poured onto minimal glucose agar plates (Kyokuto Pharmaceutical Industrial Co., Ltd., Tokyo, Japan) and incubated at 37 °C for 48 h. When the number of revertant colonies observed in the assay plate was two times higher than that of the negative control, the result was considered to be mutagenicity-positive. However, if the revertant colony generation had no dose dependence, the results were regarded as mutagenicity-negative.

In the assay, BM53-1 cell suspensions were prepared by 2-fold serial dilutions at 5000–312.5 μg/plate, and the concentrations of those mutagens were as follows: AF-2, 0.01 or 0.1 μg/plate; SAZ, 0.5 μg/plate; ICR-191, 1.0 μg/plate; 2-AA, 2.0 or 10.0 μg/plate; and B[α]P, 5.0 μg/plate.

### 2.8. RNA Extraction and qRT-PCR Analysis

Total RNA from the *S. mutans* MT8148R cell was extracted using a NucleoSpin RNA II (Macherey-Nagel GmbH & Co. KG, Düren, Germany) according to the manufacturer’s instructions. Using a ReverTra Ace qPCR RT Master Mix with gDNA Remover (TOYOBO, Osaka, Japan) enabled the residual DNA fragments to be digested, and the RNA was converted to cDNA according to the manufacturer’s instructions.

The qRT-PCR was performed on the PikoReal Real-Time PCR System (Thermo Fisher Scientific, Waltham, MA, USA) with the KAPA SYBR FAST qPCR Master Mix (2×) Universal (Kapa Biosystems, Wobum, MA, USA). The PCR was performed as follows: an initial 30 s at 95 °C, followed by 40 cycles of 5 s at 95 °C and 30 s at 60 °C. The genes *gtfB*, *gtfC*, and *gtfD* were amplified as targets using each cDNA sample as a template with the gene-specific primer sets listed in Table 1, and the relative transcriptions of the target genes were normalized to *gyrB* and used as a housekeeping gene.

### 2.9. Analyses of the GI Molecular Mass

Prior to the molecular mass and constitution sugar analyses, the GI-containing fraction was further purified based on the EPS purification procedure described previously [25]: the concentrated GI fraction prepared from the culture supernatant of BM53-1 was mixed with the same volume of acetone and incubated overnight at 4 °C. The precipitate was collected and dissolved into a 50 mM Tris-HCl buffer (pH 8.0) and treated with nuclease and proteinase K. The GI collected by ethanol precipitation was dissolved into distilled water and applied to the anion exchange resin (TOYOPEARL DEAE-650M, Tosoh Bioscience, Tokyo, Japan). The column through fraction was pooled and then dialyzed against distilled water using an Amicon Ultracel-10K ultrafiltration device.

The molecular mass of the GI was estimated with gel-filtration column (Sephacryl S-500 HR, GE Healthcare, Chicago, IL, USA) chromatography using a high-performance liquid chromatography (HPLC) system as follows: a 50 mM Tris-HCl buffer (pH 8.0) was used as a mobile phase at a flow rate of 0.5 mL/min. The eluent was divided into 95 fractions every 3 min from 0 to 285 min, and the GI content was monitored using a phenol-sulfuric acid method [37]. The molecular mass was calculated from the calibration curve made by dextran standards of known molecular weights: 25, 50, 270, and 670 kDa.

### 2.10. Analyses of Each Monosaccharide Contained in the GI

The composition of monosaccharide in the GI was analyzed via the alditol acetate derivatization method [38] using gas chromatography–mass spectrometry (GC–MS) instruments as follows: for the detection of neutral sugars, 5 mg of the purified EPS was dissolved into 1 mL of 2 M trifluoroacetic acid (TFA) and hydrolyzed at 120 °C for 1 h. After adding 1 mL of isopropanol, the mixture was evaporated under a vacuum condition. The dried monosaccharide was further dissolved into 0.5 mL of 1 M ammonium water containing 20 mg of sodium tetrahydroborate and left at RT for 1 h to reduce the polysaccharide. After the reaction, the solution was neutralized with 0.1 mL of 5 M acetic acid. The remaining borate was removed by adding 1 mL of 10% (*v/v*) acetic acid in methanol and evaporating under the vacuum condition. After three repetitions of the process, the sample was mixed with 0.5 mL of a pyridine/acetic anhydride mixture (1:1, *v/v*) and left to stand at 120 °C for 20 min. The reaction mixture was cooled on ice, and the remaining excess amount of reagents was removed by evaporating under the vacuum condition after adding 1.5 mL of water/methanol/toluene (1:4:1, *v/v*). After three repetitions of the process, the residue was mixed with 3 mL of dichloromethane/water (2:1, *v/v*), and the dichloromethane fraction was collected. The fraction was dried and finally dissolved into an appropriate volume of acetone.

Each derivative was analyzed with a DB-WAX capillary column (0.25 mm × 0.25 μm × 30 m) (Agilent, Santa Clara, CA, USA) using a JMS-T100GCV AccuTOF GCv 4G gas chromatograph high-resolution time-of-flight mass spectrometer (JEOL, Tokyo, Japan) equipped with a source of ions for electron ionization (EI). GC was performed under the following conditions: split injection mode (50:1), 1 μL injection, injection port temperature 230 °C, and column oven temperature programmed from 50 to 230 °C at 10 °C/min. MS was performed under the following conditions: electron ionization mode (EI positive, ionization energy 70 eV, ionization current 300 μA), ion source temperature 280 °C, and *m*/*z* range 29–800. The standard monosaccharides were derivatized and analyzed in the same manner as samples. Each peak was confirmed by comparing the retention time and MS with those of the standards, consisting of glucose, xylose, arabinose, mannose, galactose, rhamnose, ribose, and fucose.

## 3. Results

### 3.1. Isolation of the GI-Producing LAB Strain

**Figure 1:**
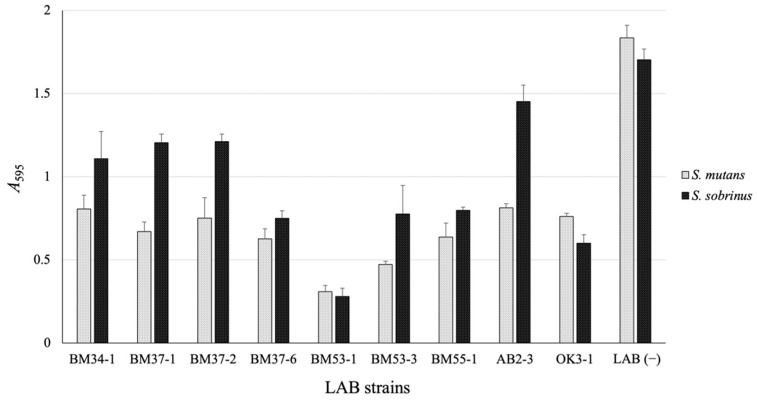
The sticky glucan production by *S. mutans* or *S. sobrinus* that have or have not been incubated with members of the LAB library. After the co-cultivation of LAB with *S. mutans* MT8148R or *S. sobrinus* ATCC27607, the formed glucan was stained and calculated using the crystal violet method. The data are indicated as means of independent assays with a standard error. LAB (−), only *S. mutans* or *S. sobrinus* was cultivated without LAB.

To screen for the LAB strain inhibiting formation of the biofilm material, many LAB strains stored in our library were evaluated using *S. mutans* MT8148R or *S. sobrinus* ATCC27607 as a test organism, and the amount of glucans produced by the test bacterium was compared with that in the absence of LAB. After screening, nine LAB isolates were selected as candidates that have anti-biofilm-forming activity (Figure 1), and the BM53-1 strain, which was isolated from the *Actinidia* (*A*.) *polygama* flower, strongly inhibited the *Streptococcus*-derived glucan formation. Based on the entire 16S rDNA sequence analysis, the BM53-1 strain was identified as *Lb. reuteri*.

Considering the application in the food industry, we tried to use edible fruit or vegetable juices as a culture medium instead of MRS broth. Finally, we employed carrot juice for BM53-1 strain cultivation. Although the GI activity is obviously maintained in the carrot juice medium, the unfermented carrot juice promoted the glucan formed by *S. mutans* MT8148R (Figure S1), demonstrating that the GI is not contained in carrot juice.

### 3.2. Confirmation of the Bactericidal Activity of the BM53-1 Strain

**Figure 2:**
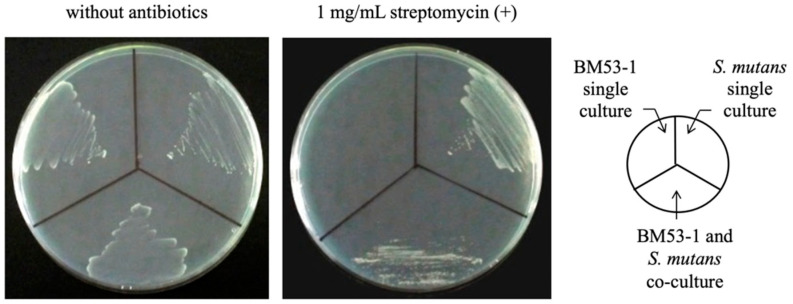
Confirmation of living *S. mutans* cells after co-cultivation with *Lb. reuteri* BM53-1. The aliquot of co-cultured broth was plated onto MRS agar with or without 1 mg/mL streptomycin. The single cultured broth of each strain was also plated for the control.

Prior to the purification of the GI, we confirmed that the BM53-1 strain inhibits the production of *S. mutans-*derived sticky glucans by whether or not there was antibacterial activity against *S. mutans* MT8148R. Although *S. mutans* MT8148R is resistant to streptomycin, the BM53-1 strain is not. After a co-culture of both bacteria, an aliquot of the culture broth was plated on MRS agar with or without streptomycin (Figure 2). The living cells on the streptomycin-containing medium were *S. mutans* MT8148R. In fact, the MT8148R strain was able to grow in the medium added with the culture supernatant of BM53-1, suggesting that *Lb. reuteri* BM53-1 did not produce the antibacterial substance against the *S. mutans* MT8148R strain (Figure S2). The result demonstrates that the BM53-1 strain produces a substance that inhibits the *S. mutans-*derived sticky glucan synthesis without the growth inhibition.

### 3.3. Safety Evaluations of BM53-1 for Food Manufacture

Through the acute oral toxicity examination using rats with oral administration of the BM53-1 culture broth, no administration-dependent illness or death was observed. Furthermore, no significant changes of the body weight and no abnormal activity, behavior, and health status of the rats were observed.

Generally, chemical substances are sometimes converted into mutagen during the metabolism process. Therefore, the S9 mixture, which is the enzyme mixture and can metabolize chemical substances, has been used to assess the mutagenic potential of the substances [39]. Regardless of the presence or absence of S9 mixture, no significant increment of the number of revertant colonies and dose-dependent manner were observed through the bacterial reverse mutation test on the BM53-1 strain, showing that the strain does not display the mutagenicity risk.

### 3.4. Effect on the GTF Gene Expression of S. mutans

**Figure 3:**
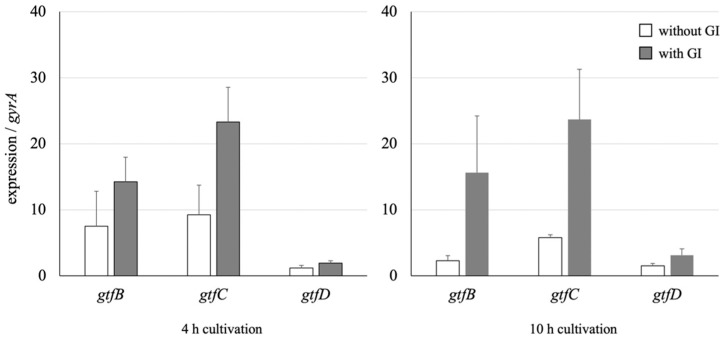
Expression differences of three gtf genes with or without the GI fraction. The data were corrected from the *S. mutans* cells cultured in the absence or presence of a GI fraction. The results were normalized to the housekeeping gene (*gyrA*). The data are indicated as means of independent assays with a standard error.

The qRT-PCR method was used to analyze whether the expression of three GTF genes, *gtfB*, *gtfC*, and *gtfD*, during the culture of *S. mutans* was altered in the presence or absence of the GI (Figure 3). The expressions of *gtfB* and *gtfC* were enhanced at an early stage (4 h) of cultivation (1.9- and 2.5-fold, respectively) in the presence of the GI. Although the enhanced expression levels of both genes were maintained after further cultivation (10 h) with the GI, those of *gtfB* and *gtfC* decreased 3.2- and 1.6-fold in the absence of the GI. In addition, the *gtfD* expression level was not significantly affected in the presence of the GI. The *comCDE*, *comS*/*comR*, and *sigX* expressions, which have been reported to participate in the quorum-sensing signal-transduction system [40,41,42], did not change (Figure S3).

### 3.5. SEM Analyses of the Morphology of S. mutans Cells in the Presence or Absence of the GI

**Figure 4:**
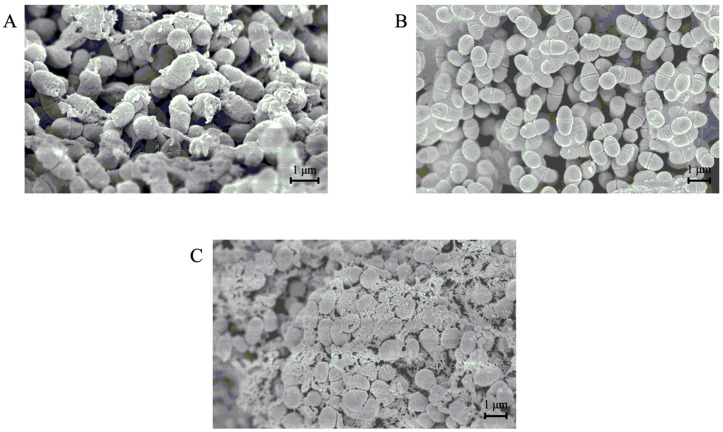
SEM images of *S. mutans* (magnification 12,000×). SEM images were captured after the cultivation of *S. mutans* in the absence (**A**) or presence (**B**) of a GI fraction. Additionally, the image was captured when *S. mutans* was cultured in the medium supplemented with an unfermented carrot juice medium (**C**). The observed glucans on the cell surfaces (**A**) were eliminated by adding the GI (**B**), and the unfermented carrot juice medium seemed to enhance glucan production.

To observe the extracellular and insoluble glucans produced by *S. mutans*, SEM images of the bacterium were captured after cultivation with or without the GI (Figure 4). When *S. mutans* was cultured in the presence of sucrose, the produced glucan was observed on the surface of the bacterium, as shown in Figure 4A. Significantly, the glucan production was decreased by adding the GI, and the cell surface was smooth (Figure 4B). *Streptococcus mutans* cells produced glucans at a high level and aggregated to each other in the presence of an unfermented carrot juice medium (Figure 4C). This observation is consistent with the result that the unfermented medium promoted the formation of glucans as a biofilm material.

### 3.6. Characteristics of the GI Prepared Using the Exopolysaccharide (EPS)-Purification Method

The averaged molecular mass of the GI purified from the supernatant of BM53-1 cultured in a carrot juice medium, which was estimated based on a gel-filtration HPLC profile, was approximately 30 kDa. On the other hand, that of the water-soluble polysaccharide detected originally in the unfermented carrot juice medium (WSP_unf_) was about 150 kDa.

We carried out a kinetic analysis to evaluate the biofilm-inhibitory effect on the GI by calculating the IC_50_ value, which is defined as the concentration inhibiting 50% of the glucan formation by *S. mutans*. The IC_50_ value (73 μg/mL) is remarkably lower than that of the WSPunf (<10,000 μg/mL).

**Figure 5:**
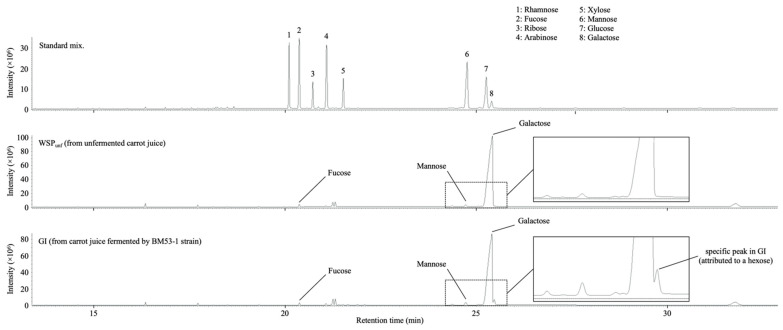
Chromatographic profiles of GC–MS analysis of the GI from the fermented carrot juice medium with the BM53-1 strain. The component monosaccharides are detected as alditol acetate derivatives. The identity of each peak was confirmed by its retention time of standards and mass spectrometry. The dashed squares are enlarged in the right panel.

The GC–MS profile for the GI indicates that the sample consists of mainly galactose and slight amounts of fucose, arabinose, and mannose (Figure 5). The few minor unattributed peaks are also predicted to originate from monosaccharide. Although the same peaks can be observed in the profiles for the WSP_unf_, only one peak at approximately 25.5 min from the GI is specifically detected and attributed to a hexose, except for the sugars used as standards.

GC–MS analysis of amino sugars, *N*-acetylglucosamine, *N*-acetylgalactosamine, and *N*-acetylmannosamine, was also performed, but attributed peaks were undetectable in all samples (Figure S4).

## 4. Discussion

Dental caries is the most prevalent infectious disease and has spread worldwide with the consumption of sugar-containing foods [43]. The disease is caused by a biofilm material-forming cariogenic bacteria, such as *S. mutans*. For the treatment of infectious disease, antibiotics have been prescribed since the middle of the 20th century and have brought a reduction in infection-associated illness and death. However, the overuse of antibiotics has led to the emergence of drug-resistant pathogens. Since antibiotics also kill some bacteria that promote host immune function and health, dysbiosis of the gut and oral microbiome occurs [44]. The present study revealed that a plant-derived lactic acid bacterial strain, designated BM53-1, produces a water-soluble polysaccharide that inhibits the production of sticky β-glucans by *S. mutans*.

Three GTF genes, *gtfB*, *gtfC*, and *gtfD*, are involved in the sticky glucan biosynthesis by *S. mutans* and play different roles in the glucan formation [12,45,46,47,48,49]. GtfB and GtfD synthesize mainly α-1,3-linkaged insoluble and α-1,6-linkaged soluble glucans, respectively. On the other hand, the glucans produced by GtfC are composed of α-1,3- and α-1,6-linkages, and the GtfC-derived glucan itself is insoluble and sticky. At the beginning of glucan biosynthesis, GtfC and GtfD cooperatively synthesize the insoluble and sticky glucans functioning as foundations for *S. mutans*, which binds to the tooth surface. After that, the oral biofilm “dental plaque” is formed with many other incorporated oral microorganisms through the synthesis of large amounts of insoluble glucans from GtfB.

**Figure 6:**
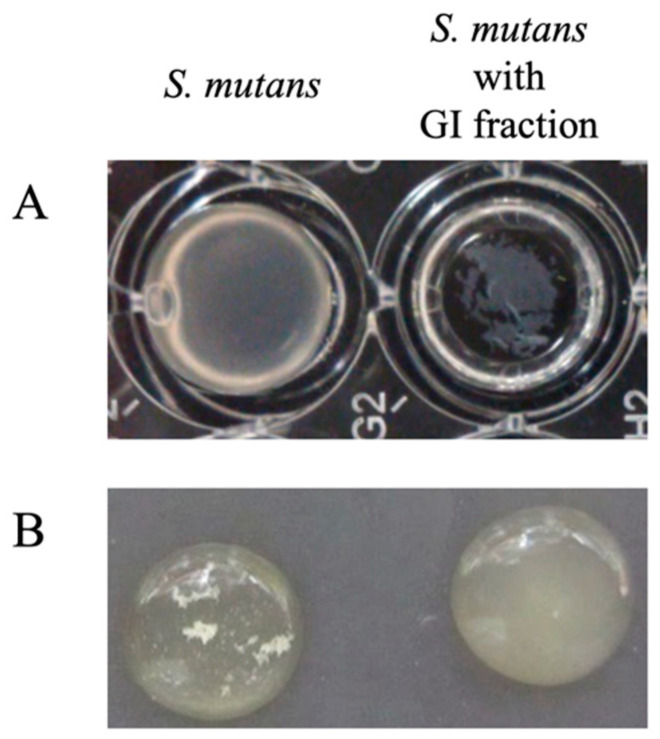
Changes in *S. mutans* cultivation by adding a GI fraction. *Streptococcus mutans* was cultured in the presence or absence of a GI fraction through static cultivation using a 96-well plate (**A**) or shaking cultivation using test tubes (**B**). The GI fraction inhibited *S. mutans* from generating the biofilm-like solid substance on the bottom of a 96-well plate, and cell aggregation of the bacterium was also eliminated by adding the GI fraction in the case of shaking cultivation.

In this study, we found that a plant-derived *Lb. reuteri* BM53-1 produces an extracellular substance that inhibits the sticky glucan production by *S. mutans*. Judging from the result of qRT-PCR analysis, the expression levels of *gtfB* and *gtfC* were obviously more enhanced than that of *gtfD* in the presence of the GI, indicating that the amount of GtfD enzymes, which are necessary to give stickiness to insoluble glucans, was relatively decreased. Therefore, it was suggested that the GI did not decrease the production of insoluble glucans but reduced the viscosity and changed the characteristics of the produced glucans. The resultant glucans seem to no longer play a role in cell adhesion and aggregation, with the result that biofilm formation was inhibited in the presence of the GI. This hypothesis is consistent with the following observations on *S. mutans*: the biofilm-like precipitant formed in a 96-well plate was easily collapsed, and the cell aggregation was repressed in a shaking culture (Figure 6).

The predicted biosurfactant-like compound produced by *Lb. acidophilus* DSM20079 has been reported to inhibit the biofilm formation of *S. mutans* through the repression of *gtfB* and *gtfC* expression [50]. It has also been reported that *Lb. brevis* KU15153 and two strains of *Lb. salivarius*, named as K35 and K43, possess bactericidal activity against *S. mutans*, resulting in biofilm-forming inhibition [51,52]. In addition, both *Lb. salivarius* strains have the ability to downregulate *gtfB*, *gtfC*, and *gtfD* genes [51]. However, there are reports that the *gtfB* and *gtfC* genes, but not the *gtfD* gene, in the *luxS*-null mutant of *S. mutans* were upregulated in the mid-log growth phase, resulting that the biofilm formation by the *luxS*-null mutant was markedly attenuated compared to the parental strain [48,49]. Those studies indicate that the imbalance in expression of three *gtf* genes results in a loss of biofilm-forming phenotype.

To confirm whether the fermentation process is necessary for production of the GI, we also purified the WSP_unf_ from unfermented juice using the same method. The GI certainly inhibited glucan production, but the WSP_unf_ did not. The chromatographic profile of the GC–MS analysis of both compounds showed that the specific peak attributed to a hexose was detected only in the GI (Figure 5). Furthermore, the averaged molecular mass of the GI was calculated as 30 kDa, which is 5-fold lower than that of WSP_unf_. Judging from those results, the WSP_unf_ contained in the unfermented carrot juice medium may be partially modified and degraded into smaller units during the BM53-1 fermentation. Interestingly, the anti-glucan-synthesis activity of the GI was resistant to heat treatment at 100 °C for 10 min, but proteases, such as proteinase K, partially decrease the inhibitory activity (Figure S5), suggesting that the GI contains a peptide linkage that is important for the GI activity.

It is still unclear why the expressions of *gtf* genes were altered. Recently, a novel signal transduction cascade named the *comS*/*comR* system was reported in *S. mutans* [40]. The signal peptide called XIP (*sigX* inducing peptide), a precursor peptide that is encoded by *comS*, is secreted to the medium and then incorporated into the bacterial cell to activate the ComR. The Rgg-like regulator ComR positively regulates the sigma-factor encoding gene *sigX* (also called *comX*), which is necessary for the genetic competence of *S. mutans*. There are some reports on the relationship between XIP and *S. mutans* showing that (1) the addition of XIP to the medium during cultivation eliminates the biofilm-forming activity and causes a decrease in the expression of *gtfB*, *C*, and *D*; (2) the loss of biofilm depends on the presence of *comR*; and (3) the expression levels of the three *gtf* genes are enhanced 10–40 times higher in the *comR* or *comX* gene-disrupted mutants than in the parental strain [42,53,54]. Based on these reports, we investigated whether the *comS*/*comR* system participates in the changes in *gtf* expressions observed in the present study; however, the GI fraction did not notably affect the *comS*/*comR*-related gene expressions, indicating that changes in the sticky glucan synthesis caused by the BM53-1 strain seem to be due to other mechanisms. How does the GI cause the imbalance of *gtf* gene expressions? Research to answer this question is now in progress.

Our recent reports have shown that some EPSs produced from the plant-derived LAB strains have a hyaluronidase-inhibitory effect [55,56,57], which has been found to correlate with the inhibition of histamine-mediated inflammatory reactions [58,59,60]. In fact, one of the EPSs produced by *Lb. paracasei* IJH-SONE68 has been reported to display prevention effect in picryl chloride-induced contact dermatitis model mice [25]. Furthermore, a clinical trial in subjects with allergic conditions using the IJH-SONE68 strain is in progress (UMIN Clinical Trials Registry, trial No. UMIN000036317, http://www.umin.ac.jp/ctr/index.htm, accessed on 16 May 2021). Therefore, although the GI is predicted to be a relatively short polysaccharide with an average molecular weight of 30 kDa, the GI may have an anti-inflammatory effect as other EPSs we have previously reported.

For an industrial application of the GI to develop the oral health-care products, there are still some issues that we have to settle. Human saliva consists of mostly water and other secreted proteins, such as enzymes and immunoglobulins, to aid digestion of foods and protection from infectious disease [55]. As described above, because the GI is found to contain a peptide linkage, the protease sensitivity of the GI should be considered when the commercial use is assessed. The development of new products using the BM53-1 strain has been undertaken through the industry–academia collaborative research, thus in vivo effectiveness of the GI will be confirmed by the animal experiment using the *S. mutans*-infected model mice. The clinical trial will also be planned in the near future for the evaluation of preventive effect for dental caries and periodontal inflammation.

## 5. Conclusions

Although many kinds of bacteria are involved in the occurrence and development of dental caries, *S. mutans* is regarded as the major pathogen of the disease [61,62,63]. *Lactobacillus reuteri* BM53-1 produces a substance that causes the stickiness of glucan to disappear, through unbalance of the expression levels of three *gtf* genes of *S. mutans*.

## Figures and Tables

**Table 1:** Primers used in this assay.

Name	Sequence (5′→3′)	Target
*gtfB*-F	ACTTTCGGGTGGCTTGGTT	*gtfB*
*gtfB*-R	GCTTAGATGTCGCTTCGGTTG	*gtfB*
*gtfC*-F	CCATGACAGTGAAGTGCAGGA	*gtfC*
*gtfC*-R	CCATAGTGAAAGAATACCCGACAAC	*gtfC*
*gtfD*-F	AAGGCGGTGCTCTGCTTTAT	*gtfD*
*gtfD*-R	ACTGGTTGGTGTGCGGTTC	*gtfD*
*gyrA*-F	ACAGAAGCGCGTATGAGCAA	*gyrA*
*gyrA*-R	TGTCGCTCCATTAACCAGAAGA	*gyrA*

## Data Availability

The data presented in the study are available in article.

## Supplementary Materials

The following materials are available online at https://www.mdpi.com/article/10.3390/microorganisms9071390/s1, Figure S1: The sticky glucan production by *S. mutans* MT8148R that have been cultured with unfermented or BM53-1-fermented carrot juice. Figure S2: *Streptococcus mutans* MT8148R culture broth in the presence of the culture supernatant of BM53-1 through shaking cultivation using a microcentrifuge tube. Figure S3: Expression differences of genes that have been reported to participate in the quorum-sensing signal-transduction system with or without the GI fraction. Figure S4: Chromatographic profiles of GC–MS analysis for amino sugars of the GI from the fermented carrot juice medium with the BM53-1 strain. Figure S5: The sticky glucan production by *S. mutans* MT8148R that have been cultured with GI fraction treated with heat, proteinase K, or trypsin.
