# Supplementary material for: Lactobacillus reuteri BM53-1 Produces a Compound That Inhibits Sticky Glucan Synthesis by Streptococcus mutans
Source: Microorganisms. 2021 Jun 27;9(7):1390. doi: 10.3390/microorganisms9071390 (PMC8307965; doi:10.3390/microorganisms9071390)
Supplement: Supplementary file 1 [file microorganisms-09-01390-s001.zip › microorganisms-1242875-supplementary.pdf]

Supplementary Figures

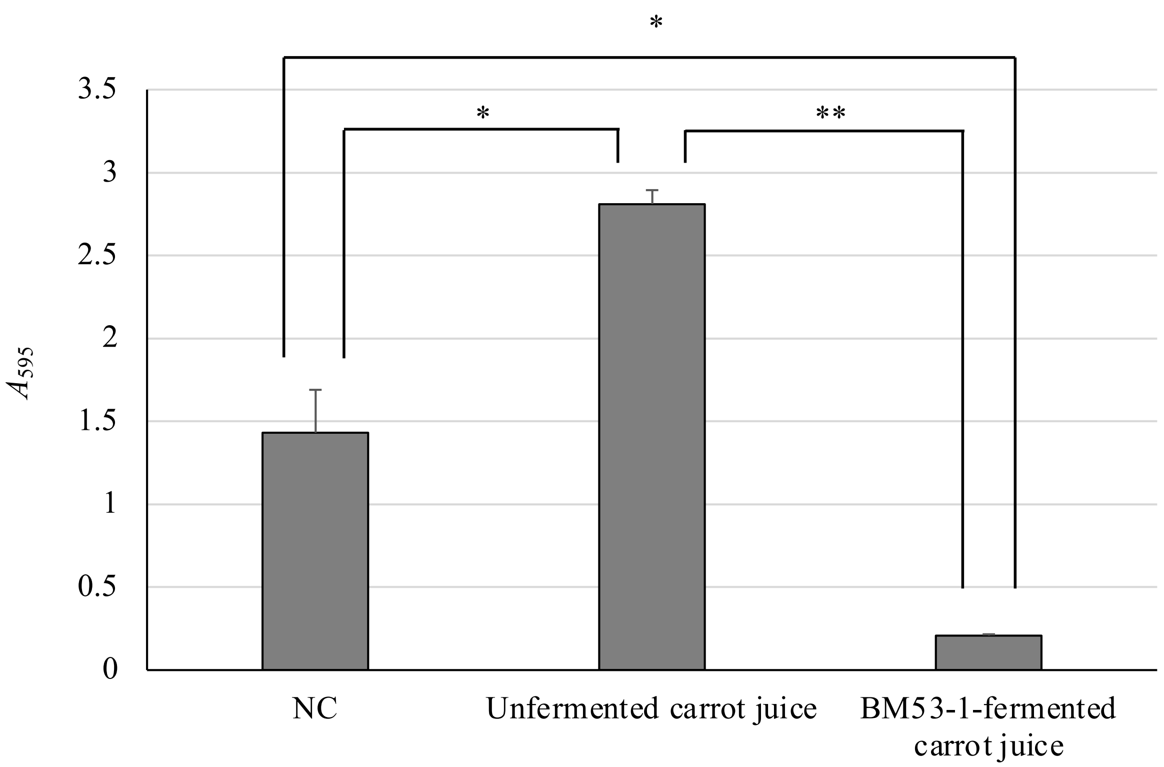

Figure S1. The sticky glucan production by *S. mutans* MT8148R that have been cultured with unfermented or BM53-1-fermented carrot juice. After cultivation, formed glucan was stained and calculated using the crystal violet method. The data are indicated as means of independent assays with a standard error. The statistical analyses were performed using the Tukey–Kramer multiple comparison test (*, p<0.05; **p<0.01).

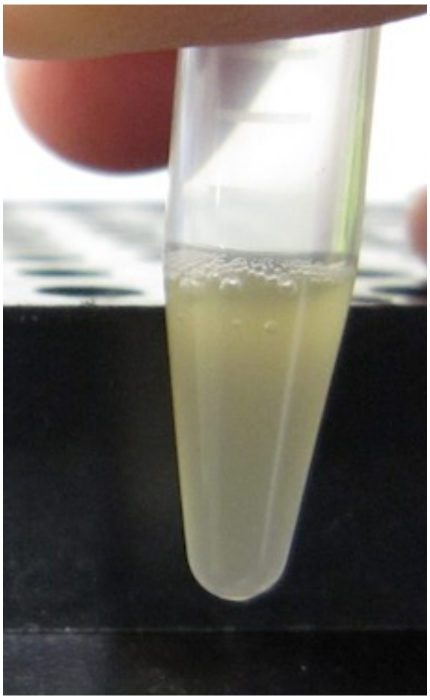

Figure S2. *Streptococcus mutans* MT8148R culture broth in the presence of the culture supernatant of BM53-1 through shaking cultivation using a microcentrifuge tube.

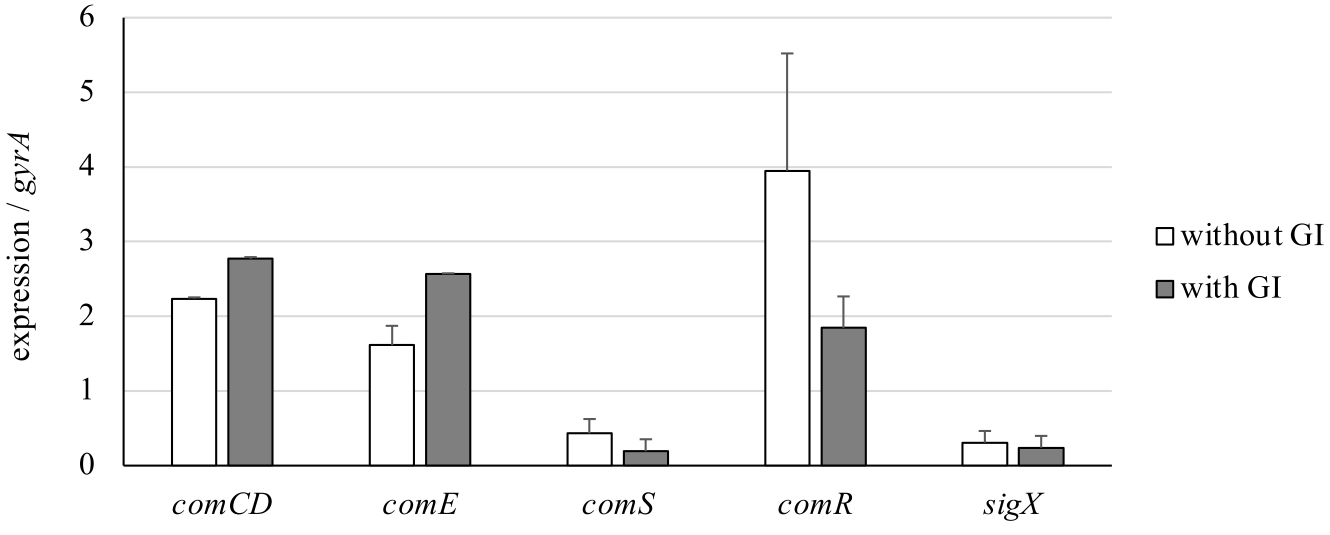

Figure S3. Expression differences of genes that have been reported to participate in the quorum-sensing signal-transduction system with or without the GI fraction. The data were corrected from the *S. mutans* cells cultured in the absence or presence of a GI fraction after 10 h cultivation. The results were normalized to the housekeeping gene (*gyrA*). The data are indicated as means of independent assays with a standard error.

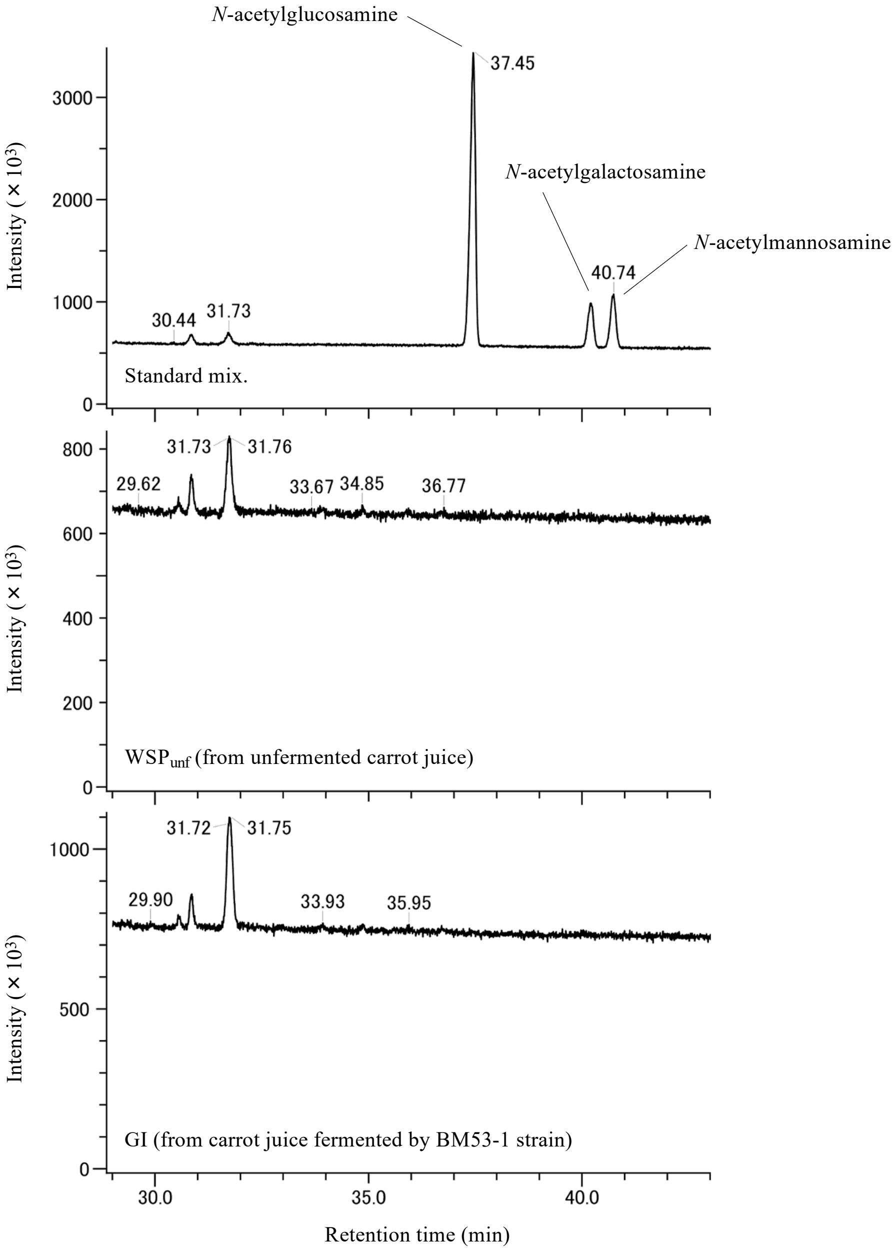

Figure S4. Chromatographic profiles of GC–MS analysis for amino sugars of the GI from the fermented carrot juice medium with the BM53-1 strain. The component monosaccharides are detected as alditol acetate derivatives. The identity of each peak was confirmed by its retention time of standards and mass spectrometry.

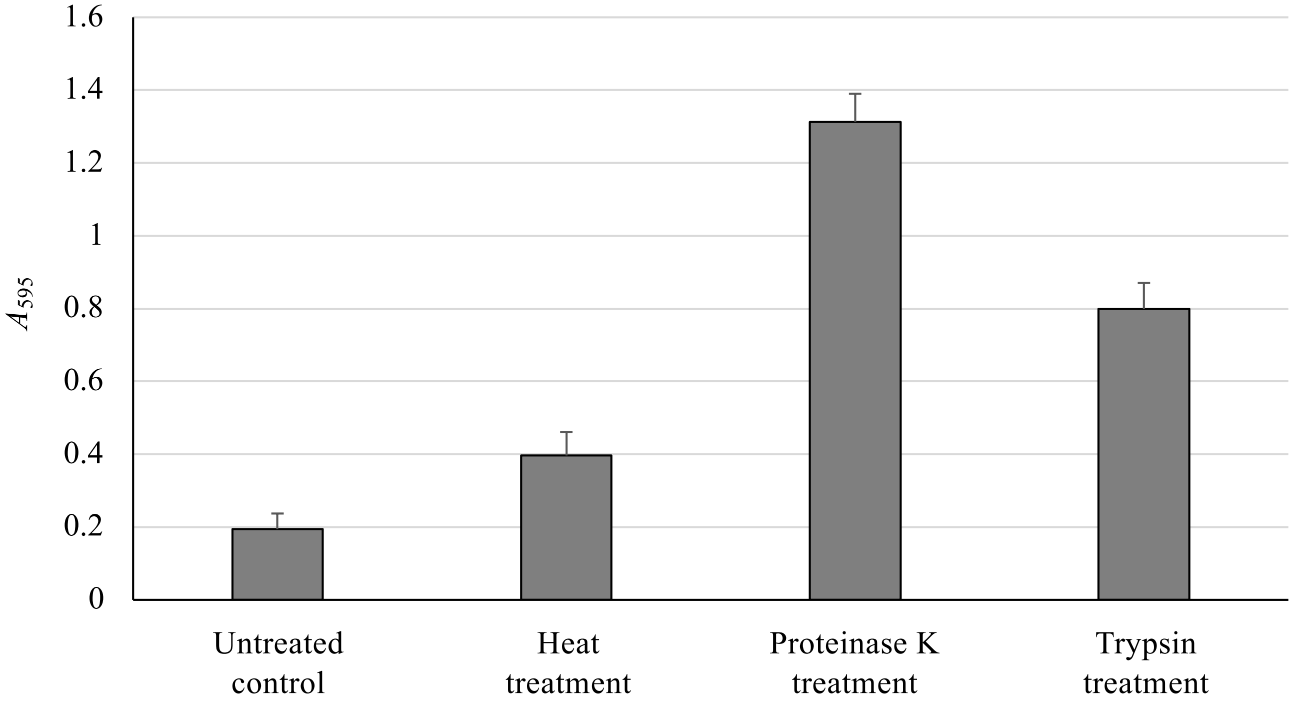

Figure S5. The sticky glucan production by *S. mutans* MT8148R that have been cultured with GI fraction treated with heat, proteinase K, or trypsin. After cultivation, formed glucan was stained and calculated using the crystal violet method. The data are indicated as means of independent assays with a standard error.
